# Omalizumab is effective in the treatment of difficult-to-treat chronic spontaneous urticaria

**DOI:** 10.1186/1710-1492-10-S2-A8

**Published:** 2014-12-18

**Authors:** Jennifer Forgie, Stephanie Santucci, Diana Pham, Genevieve Gavigan, Melanie Pratt, Simone Fahim, John O’Quinn, William H Yang

**Affiliations:** 1 Allergy and Asthma Research Centre, Ottawa, ON, Canada; 2 Division of Dermatology, University of Ottawa, ON, Canada; 3 University of Ottawa Medical School, Ottawa, ON, Canada

## Background

Chronic spontaneous urticaria (CSU) is a condition, lasting at least 6 months, where patients experience frequent episodes of red, itchy hives and/or angioedema with no apparent external trigger. For approximately 30-50% of patients this condition can resolve spontaneously but has been known to persist for years. CSU can have a major impact on a patient’s quality of life as it can affect daily activities, sleep, emotional wellbeing and social interactions. In March 2014, omalizumab was approved in Europe and eight other countries for the treatment of CSU in patients with inadequate response to H_1_-antihistamines at approved doses. However, as yet there is no approved indication for its use in CSU in Canada and the US. We report on the effectiveness of omalizumab as a treatment option for difficult-to-treat CSU in our clinic.

## Methods

After receiving a diagnosis of CSU with inadequate response to H_1_-antihistamines, and oral prednisone, patients completed a quality of life (QoL) questionnaire prior to beginning treatment with omalizumab. Patients were requested to complete the QoL questionnaires every two weeks throughout the treatment and, in addition, were monitored closely for clinical response.

## Results

A total of 10 patients, who started on omalizumab for CSU were evaluated. All were taking H_1_-antihistamines prior to treatment with 8 out of the 10 patients able to decrease or stop the use of H_1_- antihistamines after the 3^rd^ dose of omalizumab.

The results of the questionnaires indicated a 15% improvement in QoL with an accompanying 18% decrease in the symptom score. Of the 10 patients, 9 indicated an overall improvement in their symptoms while only 6 had an overall improvement in their QoL.

## Conclusion

Omalizumab is an effective therapy in difficult-to-treat CSU in our tertiary community based allergy and asthma clinic.

